# Molecular prevalence, subtype distribution, and zoonotic potential of *Blastocystis* sp. in wild rodents and shrews inhabiting Zhejiang province of China

**DOI:** 10.3389/fvets.2024.1427490

**Published:** 2024-07-02

**Authors:** Jiayan Wang, Yiqing Wang, Wenwen Huang, Ting Zhang, Kuai Yu, Jiani Chen, Liyuting Zhou, Wenjie Cao, Junchen Xu, Jianshe Ma, Huicong Huang, Wei Zhao

**Affiliations:** School of Basic Medical Sciences, Wenzhou Medical University, Wenzhou, China

**Keywords:** *Blastocystis*, molecular detection, wild rodent, shrews, zoonotic, public health, China

## Abstract

**Introduction:**

Globally, rodents and shrew populations constitute crucial elements of diverse environments and animal communities. It is imperative to study their population dynamics to mitigate any potential negative impact on humans, as they can be involved in the transmission of critical zoonotic agents, such as *Blastocystis*. Therefore, this study aimed to identify the prevalence and genetic composition of *Blastocystis* in wild rodents and shrews residing in the Zhejiang provinces of China.

**Methods:**

A total of 652 wild rodents and and shrews were captured from three different regions in Zhejiang Province from April 1st to October 31, 2023. The DNA was isolated by collecting fresh feces from the intestines of each rodent or and shrew. Rodent and shrew species were examined by vertebrate cytochrome b (*cytb*) analysis and PCR amplification. *Blastocystis* was also found in all fecal samples using PCR analysis and sequencing of the partial small subunit of ribosomal RNA (*SSU rRNA*) gene.

**Results:**

Among all the samples, 6.6% (43/652) showed a positive result for *Blastocystis*. In the results, 6 species of rodent and shrew were identified with *Blastocystis*, including *Apodemus agrarius* (*n* = 36) (2.8%), *Niviventer confucianus* (*n* = 75) (17.3%), *Rattus losea* (*n* = 18) (5.6%), *R. norvegicus* (*n* = 155) (2.6%), *R. tanezumi* (*n* = 86) (3.5%), and *Suncus murinus* (*n* = 282) (7.4%). The existence of 6 *Blastocystis* subtypes, ST4 (*n* = 33), ST1 (4), ST7 (*n* = 3), ST2 (*n* = 1), ST3 (*n* = 1), and ST5 (*n* = 1), were confirmed by sequence analysis.

**Discussion:**

Based on the molecular data obtained, the wild rodents and shrews under investigation were found to be concurrently infected with zoonotic subtypes of *Blastocystis*, including ST1 to ST5 and ST7. This suggests that these animals could potentially pose a zoonotic threat to humans and other animals susceptible to *Blastocystis* infection.

## 1 Introduction

*Blastocystis* is an anaerobic eukaryotic protist that is the only member of the stramenopiles phylum that cause infection in humans (1). More than 1 billion people worldwide are infected with this parasite, making it probably the most common intestinal parasite in humans (2). However, *Blastocystis* is often detected in asymptomatic individuals, casting doubt on its pathogenicity. Nevertheless, it is increasingly being recognized as a crucial element of the healthy gut microbiome (3). Immunocompromised patients are more vulnerable to its infection and associated symptoms like gastrointestinal distress and/or urticaria (4, 5). Meanwhile, *Blastocystis* has been demonstrated to inhabit a diverse array of animals globally, including both domestic and wild species, suggesting the likelihood of zoonotic transmission (6). Contaminated water has also been suggested as a source of *Blastocystis* infections, as evidenced by reports of its presence in surface, irrigation, and sewage water (7). *Blastocystis* sp. has been recognized as a waterborne pathogen by the World Health Organization (WHO), and it has also been designated as a prevalent eukaryotic organism in the WHO guidelines for controlling the quality of drinking water (8). Therefore, precise identification of the infection source and derived transmission pathways are crucial steps in the prevention and control of *Blastocystis* infection.

Molecular PCR-based diagnostic methods have been documented for the identification of *Blastocystis* and have demonstrated their efficacy in epidemiological studies that are actively elucidating genotype distributions across animal kingdoms and human populations (9). *Blastocystis* displays a broad genetic diversity, >40 subtypes (STs) have been described from humans and animals (10). Importantnly, 17 STs, namely ST1 to ST10, ST12 to ST14, ST16, ST23, ST35 and ST41 have been documented in humans (3, 10). These subtypes have also been identified in other mammals and birds, suggesting the possibility of zoonotic transmission (6). Even though *Blastocystis* STs do not have host specificity, ST6 and ST7 are identified most frequently in birds and infrequently in mammals, whereas ST10 and ST14 are detected most commonly in ungulates (6). Therefore, precise identification of *Blastocystis* STs is crucial for understanding zoonotic transmission, public health importance, and pathogenesis. The detection of *Blastocystis* STs in various hosts by molecular characterization is essential in understanding the transmission of this parasite.

Globally, rodents and shrews are overpopulating, particularly in some wildlife species. Their living space is closely related to human life, with extensive overlapping areas. Presently, there has been a major increase in concerns regarding the public health risks associated with wild rodents and shrews (11). As potential vectors or receptors of an already-established *Blastocystis* sp. infection in water, rodents and shrews might significantly contribute to the parasite’s distribution (12). Based on the data provided, rodents have 13 distinct STs such as ST1-ST8, ST10, ST13, ST15, and ST17, as well as several other STs that have not been named or identified (12). All *Blastocystis* STs infecting rodents, except for the ST13, ST15, ST17, and the unnamed/unknown STs, have been found in humans (3, 12). This indicates that rodents could serve as reservoirs for *Blastocystis*. Despite this understanding, significant gaps exist concerning the incidence of *Blastocystis* infection in various nations and territories. In China, only a limited number of species have been the focus of molecular examinations regarding *Blastocystis* in wild rodents or shrews (13). Therefore, it is important to increase surveillance of wild rodents and shrews to evaluate the extent of their *Blastocystis* carriage and its effects on public health.

*Blastocystis* has been observed to show a high prevalence in diverse animal species, including pigs, cattle, chickens, and horses, within the geographical region of China (14). Further, they have also been detected in children, individuals with diarrhea, patients with human immunodeficiency virus among men who have sex with men, and in the water sources of other cities (15–17). *Blastocystis* has been consistently detected in humans in Zhejiang Province, China (18, 19). However, the cause of the human infection has not been fully understood. This study explored the distribution, prevalence, and genetic composition of *Blastocystis* STs identified in wild rodents and shrews inhabiting Zhejiang Province, China due to the public health concern concerning their potential role as carriers of *Blastocystis*.

## 2 Materials and methods

### 2.1 Ethics statement

The protocols of the current study received extensive review and approval from the respective Research Ethical Committee of Wenzhou Medical University (approval number SCILLSC-2021-01).

### 2.2 Sample collection

**Figure 1:**
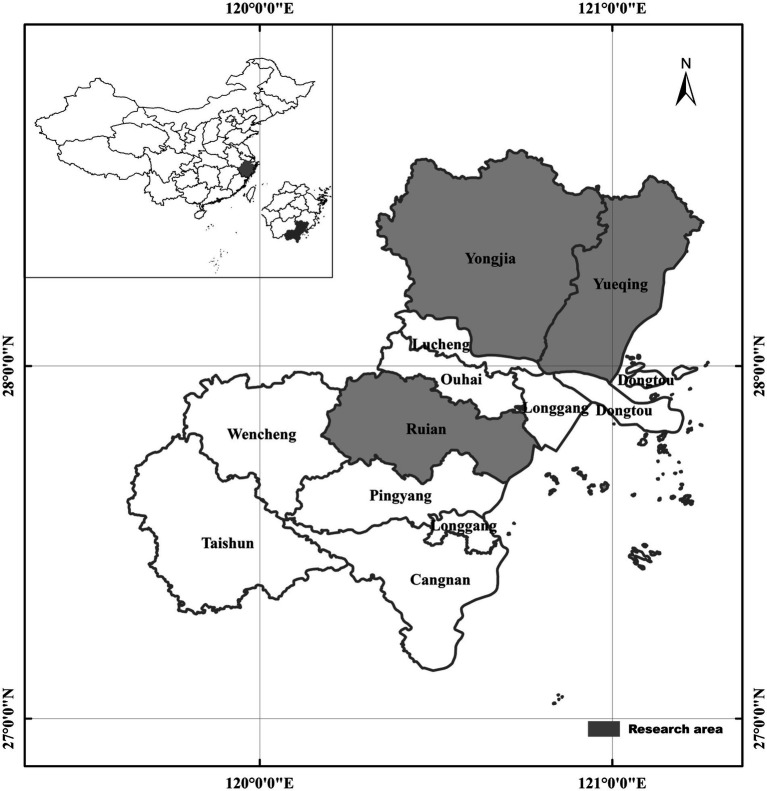
Map of rodent sampling locations in Zhejiang Province, China. The map was designed by the authors using ArcGIS 10.4 software. High-quality vector diagrams from the National Geomatics Center of China (http://www.ngcc.cn) were integrated into the design. Microsoft PowerPoint 2003 and Adobe Photoshop CS6 software were used to enrich the map with essential information.

A total of 652 wild rodents and shrews were collected from three different regions of Zhejiang Province, China from April to October 2023. These regions comprised Yongjia (170 rodents), Yueqing (94 rodents), and Ruian (388 rodents) (Figure 1). These rodents and shrews were captured in cage traps baited with deep-fried dough sticks. At each specified location, approximately 50 cage traps were set up at dusk and retrieved before dawn. The traps were arranged in a straight line, with a distance of 5 m between each trap, forming transects. All rodents were transferred to the controlled laboratory setting within 48 h after being captured and euthanized using CO_2_ inhalation. Data associated with the geographical location and collection time was recorded during rodent capturing through trapping. A sample of fresh feces (500 mg) was promptly extracted from the intestinal and rectal contents of each rodent and transported to the laboratory in ice boxes. Total DNA was extracted from these samples within a week and preserved for further analysis.

### 2.3 DNA extraction

Genomic DNA was extracted from all processed samples (200 mg) using the QIAamp DNA Mini Stool Kit (Qiagen, Germany), as per the manufacturer’s guidelines. To obtain a substantial amount of DNA, the temperature of the lysate was increased to 95°C. The DNA was reconstituted in 200 μL of AE elution buffer (provided with the kit), and stored at −20°C before PCR analysis.

### 2.4 PCR amplicons

The wild rodents and shrews were detected on the species level by amplifying a 421 bp fragment of the *cytochrome b* (*cytb*) gene from fecal DNA via PCR analysis. The primer design and PCR conditions were in line with the guidelines defined by Verma and Singh (20). To identify *Blastocystis* sp., a PCR method was used to amplify a specific region of the *SSU rDNA* gene, consisting of 500 base pairs. The primers, cycle conditions, and amplification system were consistent with the procedure described by Santin et al. (21). All PCR amplifications were performed using TaKaRa Taq DNA polymerase (TaKaRa Biology, Japan). To assure quality, negative controls devoid of DNA were incorporated into each PCR assay. The PCR results were examined by agarose (1.5%) gel electrophoresis and detected via a Gel Doc EZ UV-gel imaging system (Bio-Rad Inc., United States). Colloids were visualized via staining with GelRed (Biotium Inc., CA).

### 2.5 Nucleotide sequencing and analysis

The PCR products with specified sizes were purified and then analyzed via Sanger sequencing (Sangon Biotech Co., Ltd., China). To ensure the accuracy of the sequence, we used bidirectional sequencing and further sequencing validation of some DNA samples when needed. DNASTAR Lasergene EditSeq v7.1.0 and Clustal X v2.1 were the two tools, used to carefully edit and align each strand’s sequences. Each of the reference sequences was downloaded from the GenBank.

### 2.6 Phylogenetic analysis

To determine the genetic association between the STs of *Blastocystis* and those already stored in the genebank, a partial phylogenetic analysis was carried out by constructing a neighboring-joining tree via the Mega 7. The tree was based on the evolutionary distances determined by the Kimura-2-parameter model. The dependability of the tree was evaluated with 1,000 replicates of bootstrap analysis.

### 2.7 Statistical analyses

All presented data was examined via the SPSS software (V. 22.0, SPSS Inc., United States). To assess differences in *Blastocystis* prevalence among rodent or shrew species, regions, genders, and seasons, the chi-square test was applied to each of these variables. *p* ≤ 0.05 was considered statistical significance.

### 2.8 Nucleotide sequence accession numbers

The nucleotide sequences of *Blastocystis* sp. found during this study has been submitted to the GenBank database with the accession numbers PP211995 to PP212007.

## 3 Results

### 3.1 Study population

In this study, 5 distinct species of rodents including *Apodemus agrarius* (*n* = 36), *Niviventer confucianus* (*n* = 75), *Rattus losea* (*n* = 18), *R. norvegicus* (*n* = 155) and *R. tanezumi* (*n* = 86), and one species of shrew named as *Suncus murinus* (*n* = 282) were identified via PCR and sequencing analysis of the *cytb* gene. Samples were collected during different seasonal conditions and rates; 42.0% (274/652 summer), 30.1% (196/652 autumn), 27.9% (182/652 spring). The gender distribution of the animals revealed that 45.2% (295/652) were female and 54.8% (357/652) were male (Table 1).

**Table 1:** Prevalence and subtypes of *Blastocystis* in the investigated rodents and shrews from Zhejiang Province of China by gender, location, species and season.

Category	Positive/examined (%, 95 Cl)	Blastocystis ST (*n*)
Gender		
Female	21/295 (7.1, 4.7–10.6)	ST4 (15), ST1 (4), ST3 (1), ST5 (1)
Male	22/354 (6.2, 4.1–9.2)	ST4 (18), ST7 (3), ST2 (1)
Location		
Yueqing	8/94 (8.5, 4.4–15.9)	ST4 (7), ST5 (1)
Yongjia	10/170 (5.9, 3.2–10.5)	ST4 (7), ST1 (1), ST3 (1), ST7 (1)
Ruian	25/388 (6.4, 4.4–9.3)	ST4 (19), ST1 (3), ST7 (2), ST2 (1)
Rodent species		
*Rattus norvegicus*	4/155 (2.6, 0.1–6.5)	ST4 (4)
*Suncus murinus*	21/282 (7.4, 4.9–11.1)	ST4 (13), ST1 (3), ST7 (2), ST2 (1), ST3 (1), ST5 (1)
*Rattus tanezumi*	3/86 (3.5, 1.2–9.8)	ST4 (3)
*Niviventer confucianus*	13/75 (17.3, 10.4–27.4)	ST4 (11), ST1 (1), ST7 (1)
ApodemusAgrarius Pallas	1/36 (2.8, 0.5–14.2)	ST4 (1)
*Rattus losea*	1/18 (5.6, 1.0–25.8)	ST4 (1)
Season		
Spring	17/182 (9.3, 5.9–14.5)	ST4 (15), ST5 (1), ST7 (1)
Summer	14/274 (5.1, 3.1–8.4)	ST4 (10), ST1 (4)
Autumn	12/196 (6.1, 3.5–10.4)	ST4 (8), ST7 (2), ST2 (1), ST3 (1)
Total	43/652 (6.6, 4.9–8.8)	ST4 (33), ST1 (4), ST7 (3), ST2 (1), ST3 (1), ST5 (1)

### 3.2 Infection rates of *Blastocystis* sp

*Blastocystis* sp. was identified in 43 out of 652 fecal samples, representing a prevalence of 6.6%. The remaining species were reported with 17.3% (13/75) *N. confucianus*, 2.6% (4/155) *R. norvegicus*, 7.4% (21/282) *S. murinus*, 3.5% (3/86) *R. tanezumi*, 2.8% (1/36) *A. agrarius* and 5.6% (1/18) *R. losea* (Table 1). Interestingly, there were statistically significant variations in incidence rates of *Blastocystis* sp. among 6 species (*χ*^2^ = 20.657, df = 5, *p* = 0.001). The highest infection rates among the three locations surveyed were 8.5% for Yueqing, 6.4% for Ruian, and 5.9% for Yongjia (Table 1). Differences in infection rates among these locations were not found to be statistically significant (*χ*^2^ = 0.715, df = 2, *p* = 0.700). Based on the seasonal collection time, the infection rate of *Blastocystis* in animals was 9.3% in spring, 5.1% in summer, and 6.1% in autumn. The infection rate of *Blastocystis* in female animals was 7.1% which was higher than in males (6.2%). However, no substantial differences were noted in the infection rates of *Blastocystis* based on gender or seasonal variations (*χ*^2^ = 0.213, df = 1, *p* = 0.645 and *χ*^2^ = 3.280, df = 2, *p* = 0.186, respectively).

### 3.3 Sequencing of PCR amplicons

The subtypes of *Blastocystis* were detected by sequencing each of the 43 PCR amplicons. Nucleotide sequence identified 6 previously known STs (ST1 to ST5 and ST7), without mix infections. Among all of the 43 samples analyzed, the ST4 was the most prevalent, resulting in 76.7% (33/43). This ST was found in all 6 rodent species included in the survey. The remaining STs had a low frequency with ST1 being identified in 4 samples, including 3 in *S. murinus* and 1 in *Ni. confucianus*; ST7 in 3 samples including 2 in *S. murinus* and 1 in *N. confucianus*; ST2, ST3 and ST5 only found in a *S. murinus*, respectively (Table 1).

Further, the composition of STs varied between different regions, including ST4 and ST5 in Yueqing, ST1, ST3, ST4, and ST7 in Yongjia, and ST4, ST1, ST2, and ST7 in Ruian. Based on the genders of the animals, ST3, ST4, and ST5 were identified in females, whereas ST2, ST4, and ST7 were found in males. During different seasons, these animals carried different STs; for example, 3 STs (ST4, ST5, ST7) in spring, 2 STs (ST1 and ST4) in summer, and 4 STs (ST2, ST3, ST4, ST7) in autumn (Table 1).

### 3.4 Genetic diversity of *Blastocystis* subtypes

Among the 43 sequences identified, 7 sequences have not been described previously, including 2 ST1 sequences (PP211995 and PP211996) revealed 99.8% sequence identity with KF285443 (human isolate from Malaysia and Thailand) and MG254565 (human isolate from Malaysia), with only 1 base difference; an ST3 sequence (PP211999) showed 99.54% sequence identity with the human isolate KX108728 originating from Malaysia with 2 bases difference; 2 ST4 sequences (PP212001 and PP212002) with 99.77% (1 base difference) and 97.42% (10 base difference) identity to OP268432 (*Rattus rattus* from Iran) and MT071884 (experimental rats from China), respectively; 2 ST7 sequences (PP212005 and PP212007), which differ by 2 (99.54%) and 5 (98.89%) bases from OR936687 (human from China) and OL514227 (chicken from China), respectively.

Further, the remaining 36 sequences, including 31 ST4, 2 ST1, 1 ST2, 1 ST5, and 1 ST, have been described previously. Approximately 31 ST4 sequences can be divided into 2 types: ST4-1 (PP212000) (*n* = 29) was completely homologous to MT071884 (experimental rat from China); ST4-2 (PP212003) (*n* = 2) had 100% identity to OP268432 (monkey from Iran) and 2 other sequences HQ641652 (primate from Spain) and OK235459 (capybara from China). The 2 ST1 sequences (PP211997) were similar and shared 1 sequence with ON932517 (horse from Colombia); ST2 (PP211998), ST5 (PP212004) and ST7 (PP212006) sequences were completely similar to KT591796 (capuchin in Mexico), OQ727482 (pig in China) and OR936687 (human in China), respectively.

**Figure 2:**
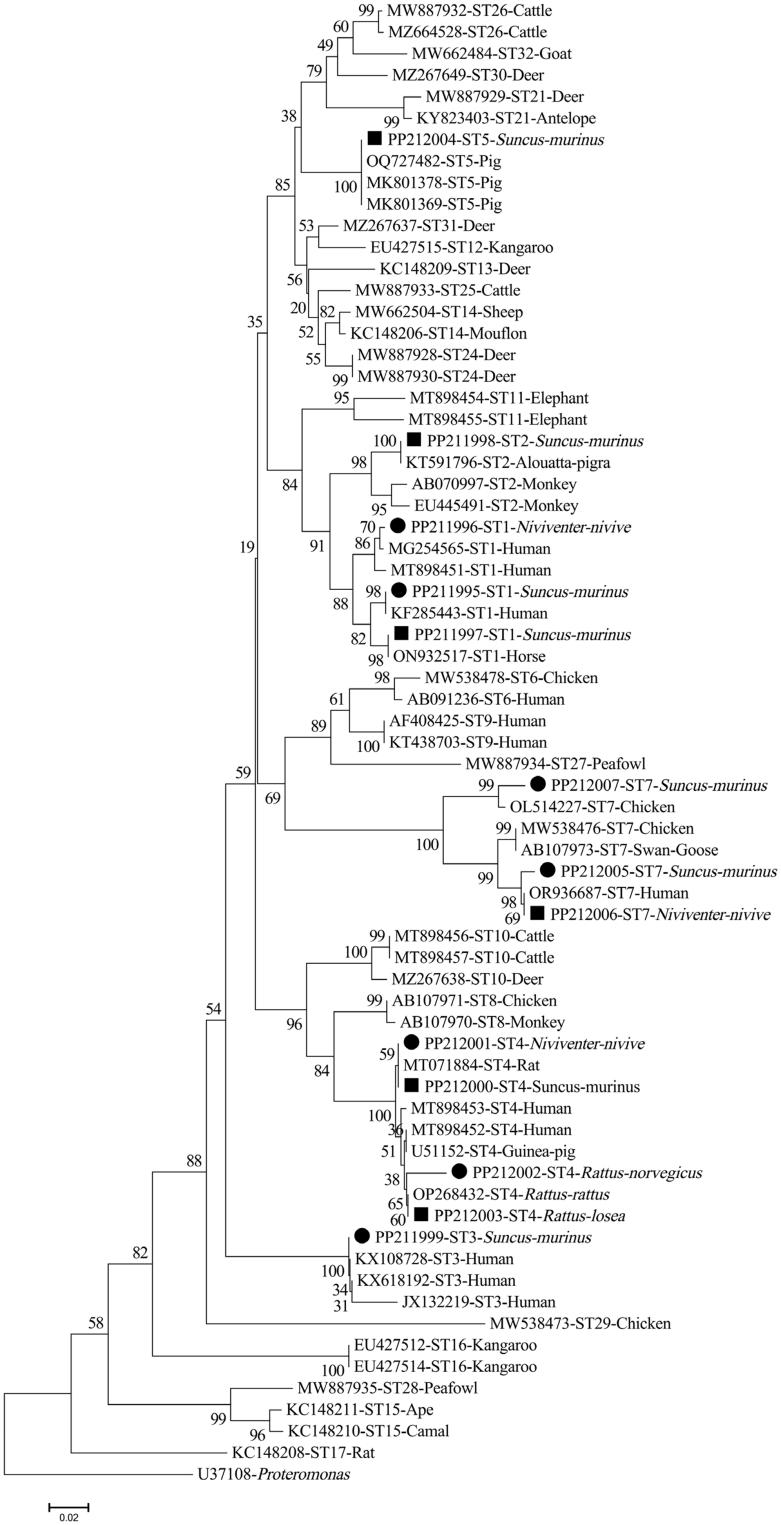
Phylogenetic tree represents the *Blastocystis* ST subtypes, based on their SSUrRNA sequences. The tree was constructed via a neighbor-joining analysis, where genetic distances were determined using the Kimura 2-parameter model. Bootstrap values (≥50%) obtained from 1,000 repeats are presented to the left of the nodes. The solid circles denote the sequences produced in the present study.

The nucleotide sequences of *Blastocystis* sp. STs discovered in the current study were grouped into the respective evolutionary branches with widely recognized STs in the phylogenetic tree (Figure 2).

## 4 Discussion

Rodents and shrews are the most abundant and diverse group of animals worldwide. Particularly wild ones are ubiquitous and their habitats widely overlap with humans and other animals. They can contaminate the environment, vegetables, fruits, and water sources by secreting infected cysts and spreading the virus to humans and other animals (11). Previous studies have confirmed that rodents widely carry *Blastocystis*, and the infection rate is up to 19.7% with wild rodents staggering 30.5% (22). *Blastocystis* is primarily found in breeding, experimental, and pet rodents in China (Table 2) (13, 23–32). There was only two study focusing on the infection of *Blastocystis* in wild rodents from China, revealing a relatively low infection rate of 3.7% in Heilongjiang Province (13) and a slightly higher rate of 37.9% in Henan Province (32). This study was the first to examine wild rodents and shrews in Wenzhou, southern Zhejiang Province, China and revealed that the average infection rate was 6.6%. Based on the current findings, the prevalence of *Blastocystis* infection in Chinese rodents was typically low (9.4%) with 7.9% for farmed, 7.7% for pet, 13.6% for wild, and 8.2% for experimental rodents (Table 2) (13, 23–32). However, the variations in infection rates may be attributed to the rodent species; for example, *Blastocystis* in *N. confucianus* was 17.3%, which was considerably higher than the rate observed in *R. norvegicus* (2.6%) in the present study. The variability in prevalence rates may be attributed to different factors such as animal species, health, sample size, environment, geographical distribution, and the type of screening methods. The infection status of *Blastocystis* in rodents may be closely related to their living environment. Regular exposure to other animals in the living environment may elevate the risk of infection due to the easier transmission of *Blastocystis*. Molecular diagnosis is currently considered an effective method for detecting and monitoring the rate of *Blastocystis* infection due to its high sensitivity and specificity (9, 33). It is important to highlight that only a single study was conducted in 12 out of the 15 countries, specifically focusing on rodents, with relatively small sample sizes (12). Thus, due to the limited data, precisely estimating the actual prevalence of *Blastocystis* infection in rodents in China or a specific region is challenging, necessitating more study for elucidation. However, this study offers fundamental data on rat *Blastocystis* infection, revealing that the wild rats harbored several STs of *Blastocystis*.

**Table 2:** Prevalence and subtypes of *Blastocystis* in the rodents from China.

Rodent types/species (Latin name)	Positive/examined (%)	STs (*n*)	References
Farmed			
Asiatic brush-tailed porcupines (*Atherurus macrourus*)	12/257 (4.7)	ST4 (11), unST (1)	(22)
Bamboo rats (*Rhizomys sinensis*)	22/480 (4.6)	ST4 (17), ST5 (5)	(23)
	8/360 (2.2)	ST4 (8)	(22)
Coypus (*Myocastor coypus*)	44/308 (14.3)	ST4 (33), ND (8), ST5 (3)	(24)
Patagonian mara (*Dolichotis patagonum*)	3/18 (16.7)	ST4 (3)	(25)
Flying squirrels (*Trogopterus xanthipes*)	21/69 (30.4)	ST13 (9), ST1 (8), ST3 (4), ST1 + ST3 (3)	(26)
Rodentia	6/33 (18.2)	ST17 (4), ST4 (2)	(27)
Masked Palm civets (*Paguma larvata*)	27/283 (9.5)	ST5 (26), ST1 (1)	(22)
Subtotal	143/1808 (7.9)	ST4 (74), ST13 (9), ST1 (9), ST5 (34), ND (8), ST3 (4), ST17 (4), ST1 + ST3 (3), unST (1)	
Laboratory			
Sprague Dawley rats	17/151 (11.3)	ST4 (16); ST7 (1)	(28)
Spontaneously hypertensive rats	3/100 (3.0)	ST4 (1); ST7 (2)	(28)
Wistar rats	9/104 (8.7)	ST4 (9)	(28)
Subtotal	29/355 (8.2)	ST4 (26), ST7 (3)	
Pet			
Chinchilla (*Chinchilla lanigera*)	3/72 (4.17)	ST4 (2), ST17 (1)	(29)
Chinese Striped Hamster (*Cricetulusbarabensis*)	12/98 (12.24)	ST4 (12)	(29)
Eurasian Red Squirrel (*Sciurusvulgaris*)	7/72 (9.72)	ST4 (7)	(29)
Eastern Chipmunk (*Tamias striatus*)	8/171 (4.68)	ST4 (8)	(29)
Guinea Pig (*Cavia porcellus*)	12/90 (13.33)	ST4 (12)	(29)
Pallas’s squirrels (*Callosciurus erythraeus*)	10/171 (5.8)	ST5 (4), ST6 (4), ST1 (1), ST3 (1)	(30)
Subtotal	52/674 (7.7)	ST4 (41), ST5 (4), ST6 (4), ST1 (1), ST3 (1), ST17 (1)	
Wild			
Asian house rat (*Rattus tanezumi*)	41/136 (30.1)	ST1 (2), ST3 (2), ST4 (8), ST5 (29)	(31)
	3/86 (3.5)	ST4 (3)	This study
Asian house shrew *(Suncus murinus)*	21/282 (7.4)	ST4 (13), ST1 (3), ST7 (2), ST2 (1), ST3 (1), ST5 (1)	This study
Brown rat (*Rattus norvegicus*)	4/108 (3.7)	ST4 (4)	(13)
	42/58 (72.4)	ST1 (1), ST2 (1), ST4 (35), ST5 (5)	(31)
	4/155 (2.6)	ST4 (4)	This study
Chinese white-bellied rat (*Niviventer confucianus*)	13/75 (17.3)	ST4 (11), ST1 (1), ST7 (1)	This study
House mouse (*Mus musculus*)	3/25 (12.0)	ST3 (1), ST4 (1), ST5 (1)	(31)
Lesser ricefield rat (*Rattus losea*)	1/18 (5.6)	ST4 (1)	This study
Striped field mouse (*Apodemus agrarius*)	1/36 (2.8)	ST4 (1)	This study
Subtotal	133/979 (13.6)	ST4 (81), ST1 (7), ST7 (3), ST2 (2), ST3 (4), ST5 (36)	
Total	357/3816 (9.4)	ST4 (222), ST5 (74), ST1 (17), ST13 (9), ND (8), ST3 (9), ST7 (6), ST17 (5), ST6 (4), ST1 + ST3 (3), ST2 (2), unST (1)	

One possible source of zoonotic *Blastocystis* sp. subgroups is wild rodents. Their ability to contain many subtypes, including over 90% of zoonotic transmission, suggests that they could be involved in the transmission of disease (12, 22). This study explored 6 known STs of *Blastocystis* sp. (ST1, ST2, ST3, ST4, ST5, and ST7), with ST4 being the most prevalent ST found in 95.7% of the animal samples. The prevalence of *Blastocystis* sp. ST4 in humans is 5.9% worldwide, whereas it represents 19.8% of reported cases in Europe (3). Previous studies have demonstrated that ST4 is found in over 19 rodent species globally; therefore, this ST may have evolved to infect rodents (12, 23). Further, it has been detected in mandrills, alpacas, artic foxes, bears, birds, buffalo, cats, cattle, deer, dogs, goats, New Zealand white rabbits, ring-tailed lemurs, pigs, and various water sources in Asia, suggesting that this subtype inhabits a wide range of hosts (1). Moreover, ST4 has been identified in humans, rodents (bamboo rats, porcupines, civets, and brown rats), bears, and whooper swans in China (13, 14, 23, 34, 35). They require full examination as they are regarded as possible carriers of human *Blastocystis* sp. infections.

Despite being found in only 0.9% (6/625) of the animals studied, the presence of ST1, ST2, and ST3 is significant due to their role as major public health infections. These three subtypes were responsible for 85.75% of all human cases (3). Meanwhile, all of them have been detected in animals worldwide (6). However, they are also widespread in Chinese animals such as ST1 is found in foxes, civets, birds, bears, non-human primates, pigs, and dogs; ST2 is observed in non-human primates, bears, and certain captive wild animals; and ST3 is identified in rex rabbits, raccoon dogs, goats, sheep, cattle, pigs, and non-human primates (13). The identification of ST1, ST2, and ST3 in the investigated wild rodents and shrews cannot be ignored, and they are highly likely to transmit their carrying *Blastocystis* to humans and other animals.

The prevalence of ST5 in human samples globally has been previously reported to be 1.64% (3). It has been found in several animal hosts, including some rodent species (6, 12, 32). The first confirmation of ST5 in *S. murinus* not only expands the understanding of the host specificity of this subtype but also suggests a potential zoonotic transmission pathway from *S. murinus* to humans, pigs, etc. It is suggested that ST5 found in *S. murinus* could have originated from pigs or humans. ST7 is the third most prevalent subtype among humans in Southeast Asian countries like Thailand, and it is frequently found in birds (1, 6). The identification of ST7 in *N. confucianus* and *S. murinus* suggests that rodents are crucially involved in the zoonotic transmission of this ST to humans.

## 5 Conclusion

This study presented the first findings regarding the occurrence and genetic variability of *Blastocystis* sp. in wild rodents and shrews from Zhejiang Province of China. The results demonstrated the presence of 6 known *Blastocystis* sp. subtypes (ST1 to ST5, and ST7) in the examined animals. Wild rodents and shrews can potentially act as a source of infection for human *Blastocystis* infection caused by the identified *Blastocystis* STs, considering these STs are already known to infect humans. To improve the understanding of the pathway by which *Blastocystis* is transmitted from wild rodents and shrews to humans, extensive research is required to explore the transmission pattern, genetic diversity, and biology of these parasites in a broader range of wild rodent and shrew species in different geographic regions.

## Data availability statement

The datasets presented in this study can be found in online repositories. The names of the repository/repositories and accession number(s) can be found in the article/supplementary material.

## Ethics statement

The animal study was approved by the respective Research Ethical Committee of Wenzhou Medical University (approval number SCILLSC-2021-01). The study was conducted in accordance with the local legislation and institutional requirements.

## Author contributions

JW: Writing – original draft, Writing – review & editing, Formal analysis, Investigation, Methodology. YW: Formal analysis, Investigation, Methodology, Writing – original draft, Writing – review & editing. WH: Formal analysis, Investigation, Methodology, Writing – original draft, Writing – review & editing. TZ: Investigation, Methodology, Writing – original draft, Writing – review & editing. KY: Investigation, Methodology, Writing – original draft, Writing – review & editing. JC: Investigation, Methodology, Writing – original draft, Writing – review & editing. LZ: Investigation, Methodology, Writing – original draft, Writing – review & editing. WC: Investigation, Methodology, Writing – original draft, Writing – review & editing. JX: Investigation, Methodology, Writing – original draft, Writing – review & editing. JM: Writing – original draft, Writing – review & editing, Resources, Supervision. HH: Supervision, Writing – original draft, Writing – review & editing, Conceptualization. WZ: Conceptualization, Supervision, Writing – original draft, Writing – review & editing, Data curation.
